# Microgravity protein crystallization

**DOI:** 10.1038/npjmgrav.2015.10

**Published:** 2015-09-03

**Authors:** Alexander McPherson, Lawrence James DeLucas

**Affiliations:** 1 University of California, Irvine, CA, USA; 2 University of Alabama at Birmingham, Center for Biophysical Sciences and Engineering, Birmingham, AL, USA

## Abstract

Over the past 20 years a variety of technological advances in X-ray crystallography have shortened the time required to determine the structures of large macromolecules (i.e., proteins and nucleic acids) from several years to several weeks or days. However, one of the remaining challenges is the ability to produce diffraction-quality crystals suitable for a detailed structural analysis. Although the development of automated crystallization systems combined with protein engineering (site-directed mutagenesis to enhance protein solubility and crystallization) have improved crystallization success rates, there remain hundreds of proteins that either cannot be crystallized or yield crystals of insufficient quality to support X-ray structure determination. In an attempt to address this bottleneck, an international group of scientists has explored use of a microgravity environment to crystallize macromolecules. This paper summarizes the history of this international initiative along with a description of some of the flight hardware systems and crystallization results.

## Introduction

The use of X-ray crystallography to determine the structures of macromolecules has progressed markedly in recent years. At one time, the analysis of the structure of a single crystalline protein might have occupied the efforts of several crystallographers for many years. Collection of X-ray diffraction data were extremely time consuming and computing procedures were similarly tedious. Few techniques existed for detailed examination of structure once it had been determined. This has changed in a striking manner. X-ray sources, both rotating anode generators and synchrotrons, have been developed that yield X-ray flux densities several orders of magnitude more intense than those available only 10 years ago. New area detector systems for the measurement of diffracted X-rays have come into use and these have had an extraordinary impact on the rate and accuracy with which X-ray data can be accumulated.^1^ What was formally a process requiring several years of intense effort has now been markedly reduced. Computing speed and precision has advanced proportionately, accompanied by the invention of mathematical procedures that greatly enhance our ability to utilize X-ray diffraction to determine and study macromolecular structure.^2^

However, the production of diffraction-quality crystals remains the major obstacle preventing determination of hundreds of important proteins. Because its value to the process was never fully appreciated, its practice considered as much art as science, the process of protein crystal growth was ignored and relegated to obscure corners of laboratories. Not only did the phenomenon of protein crystal growth remain a mystery, but its practitioners languished. Furthermore, the difficulties of growing protein crystals are many and varied. The key to overcoming this bottleneck lies in the development of new and reliable techniques, more systematic and scientific, for obtaining suitable protein crystals. This obviously includes the optimization of all relevant physical parameters. As a result of studies supported by the National Aeronautics and Space Administration (NASA) and space agencies representing several other countries, it has been clearly demonstrated that gravity effects must be included as a key crystallization parameter.

It has long been recognized for crystals grown from the melt, that mass and heat transport processes are critical determinants in the character and quality of the product. Indeed, many important crystal growth techniques have been developed to explicitly control the relative contributions of convective and diffusive transport to growth processes.^3^ Transport processes, particularly mass transport, are of importance in the growth of crystalline materials from aqueous solutions as well.^4–7^ As a consequence of the development of density differences near growing crystal surfaces, produced by active recruitment of ions or molecules from solution into the lattice, convective flow^8^ is generated in the crystal’s immediate environment.^9–15^ Transport of molecules by solutal convection competes with transport by pure diffusion, and the interaction of the two determines the modes and kinetics of the presentation of nutrient molecules to a growing crystal.

Transport affects not only the molecules or ions that are incorporated into the growing crystal, but it also affects the rate of adsorption/incorporation of impurities.^4–7^ Because impurity incorporation can have a marked impact on the size, morphological development, and ultimate perfection of a crystal, differences in form or quality produced by diversity in transport phenomena can be unexpectedly large. Impurities may be of particular significance in macromolecular systems which are marginally pure to begin with, and in addition may contain a wide variety of naturally occurring, and often unavoidable macromolecular aggregates, clusters, or oligomers.^16–23^ These impurities may be of fixed size, composition, and arrangement; they may be specific aggregates, or they may be of a more random, variable nature.

Mass transport by convection can, of course, only occur if gravity is present. Only then will heavier fluids fall and lighter fluids rise, and only then can convective currents be established in a bulk solution. There are some other kinds of convection, such as surface-tension-driven convection,^13^ but these are believed to be of little consequence for most experiments involving crystal growth from solution, but admittedly, this has received little attention.

Experience with many types of crystal growth confirmed, almost without exception, that the minimization of convective transport, which allows growth to occur principally by diffusion of molecules to the surfaces, generally resulted in crystals of superior quality with improved optical and mechanical properties, reduced defect densities, and greater size. Thus, it follows that crystals grown in a zero gravity, or a reduced gravity environment, might demonstrate enhanced properties. Because vehicles in space typically experience gravity forces of 10^−3^ to 10^−7^ *g*, this appeared to be an attractive possibility for the production of improved, perhaps even novel crystalline materials. Indeed, earlier experiments in microgravity where ionic and conventional small molecule crystals were grown supported this idea.^24–28^

### Microgravity in gravity

Vehicles orbiting the Earth do not, of course, completely escape gravitational forces. For example, the force of attraction from the Earth’s gravitational field on a satellite in high orbit is about the same as on the surface of the Earth. How then can an orbiting vehicle experience zero, or microgravity? This is particularly well explained by the following passage provided by the NASA.^29^

Many people do not realize that Earth’s gravitational field extends far out into space, in fact far beyond the orbit of the Space Shuttle. If it were possible to build a tower reaching to the height of the Shuttle’s orbit, then gravity would be almost as strong at the top of the tower as it is on the ground. A person stepping off this tower would drop to the ground, just as he or she would from a tall skyscraper. But if this is true, why do Shuttle crewmembers float and a microgravity environment exist for experiments? Sir Isaac Newton hypothesized how an artificial satellite could be made to orbit the Earth. He envisioned a very tall mountain extending above the Earth’s atmosphere so that friction with the air would not be a factor. He then imagined cannons at the top of the mountain that fired cannonballs parallel to the ground. As each cannonball was fired, it was acted on by two forces. One force, the explosion of the black powder, propelled the cannonball straight outward. If no other force were to act on the cannonball, then the shot would travel in a straight line and at a constant velocity. But Newton knew that a second force would act on the cannonball. Gravity would act to pull the cannonball down toward Earth. Because of the presence of gravity, the path the cannonball would travel would be bent into an arc ending at the Earth’s surface.

Newton’s thought experiment demonstrated how additional cannonballs would travel farther from the mountain if the cannon were loaded with more black powder each time it was fired. With each shot, the path would lengthen and soon, cannonballs were disappearing over the horizon. Eventually, a cannonball was fired with enough energy, in Newton’s imagination, that it fell entirely around the Earth and came back to its starting point. Provided that nothing would interfere with the cannonball’s motion, it would continue circling the Earth: it was in orbit.

This is how the Space Shuttle stays in orbit above the Earth. It is launched in a trajectory that arcs above the Earth so that the orbiter is traveling at just the precise speed to keep it falling while maintaining a constant altitude above the surface. For example, if the Shuttle climbs to a 320-km-high orbit, it must travel at a speed of about 27,740 km/h to achieve and maintain orbit. At that speed and altitude, the Shuttle’s falling path will be parallel to the curvature of the Earth. Because the Space Shuttle is free falling around the Earth and the friction with the upper atmosphere is extremely low, a microgravity environment is established.

### Uniqueness of macromolecular crystals

Macromolecules, being unique in their properties, both in terms of size and complexity, give rise to crystals that are also unique.^30–33^ We cannot, therefore, expect that macromolecular crystals will necessarily develop according to precisely the same mechanisms and principles as do conventional crystals.^33,34^ This is important because, if we do not know how protein and virus crystals grow, as well as understanding their detailed characteristics, then we cannot expect to understand how gravity affects the process and the quality of crystals grown in space.

Macromolecular crystals are relatively small in comparison with conventional crystals, rarely exceeding a millimeter on an edge, and generally smaller. Because only one stereoisomer of a biological macromolecule naturally exists, they do not form crystals possessing inversion symmetry, and, therefore, generally exhibit simple shapes that lack the polyhedral character of small-molecule crystals. They are extremely fragile, often crushing at the touch, degrade outside a narrow temperature, ionic strength, or pH range, generally exhibit weak optical properties,^35^ and diffract X-rays to resolutions far short of the theoretical limit.^36^ The reason for most of these character deficiencies is that macromolecular crystals incorporate large amounts of solvent in their lattices, ranging from about 30% at the lower limit to 90% or more in the most extreme cases.^37,38^ Proteins also have, as individual molecules, an array of water molecules, which surround them and are relatively tightly bound both in solution and in the crystal.^39^

There are two other crucial differences between macromolecular and conventional crystal growth that have important practical consequences. The first is that macromolecular crystals are usually nucleated at extremely high levels of supersaturation, often several 100 to a 1000%, while conventional crystals, on the other hand, usually nucleate at only a few percent supersaturation.^14^ Virtually every quantitative aspect of crystal growth is a direct function of supersaturation.^5^ Although high supersaturation may be essential to promote nucleation, it is far from ideal for growth, and the many problems observed for macromolecular crystals attest to this. Furthermore, supersaturated macromolecular solutions, in addition to crystal nuclei, produce alternate solid states that we refer to collectively as amorphous precipitates. Unlike conventional systems, competition exists at both the nucleation and growth stages between crystals and precipitate; particularly acute because competition is promoted by high levels of supersaturation. Because amorphous precipitates are kinetically favored, though of less favored energy state, they tend to dominate the equilibration process and often inhibit or preclude crystal formation.

Given the complexities that beset macromolecules, can we reasonably expect their crystallization to resemble that of conventional molecules? Evidence to this point suggests that the answer is in principle yes, but in practice, no. It appears that the fundamental mechanisms and pathways of macromolecular crystal growth are the same as for conventional crystals^40,41^ but that the magnitudes of the underlying kinetic and thermodynamic parameters that govern the process differ markedly.

Current evidence confirms that macromolecular crystals grow by the same mechanisms and reflect the same physical principles as do conventional crystals. These generally assume the generation of growth steps by two-dimensional nucleation and by spiral dislocations^5,40,41^ and pictures the ordered addition of individual molecules at the step edges at a constant rate determined by the level of supersaturation, *σ*. Disorder in the crystal, which limits diffraction resolution, is generally ascribed to the statistical spread of incorrect orientation and position of molecules from the mean. The model is made somewhat more realistic by addition of the concept of mosaicity,^36^ which describes the real crystal in terms of semiordered crystalline blocks. In general, the growth surfaces are considered to be relatively smooth, as are the step edges, with defects rather rare, though large amounts of impurities may perturb these features.

## Initial results (proof of concept)

As stated earlier, crystals grown in microgravity may demonstrate enhanced properties and vehicles experiencing microgravity are an attractive possibility for the production of improved crystalline materials. An unfortunate aspect of the research carried out in microgravity is that much of it has gone undocumented, or is described only in space agency reports, or technical notes. This is particularly true of failures or marginal successes that did not merit publication in the greater literature, or those not deemed worthy of serious treatment by the investigators. In some cases, for example, Russian and Chinese investigations, language or lack of access to international journals inhibited broad disclosure. If some surprising or exceptional results were obtained, then these possibly found their way into press, but little else. Thus, there is only an incomplete record of many of the experiments performed during the 1970s and 1980s.

**Figure 1:**
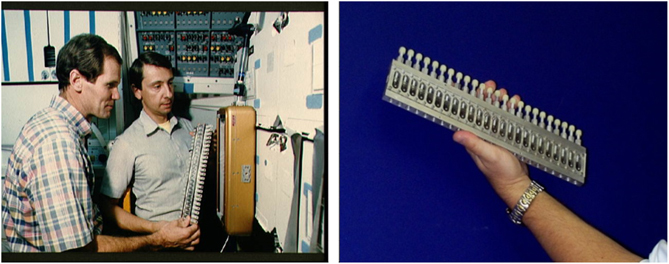
Astronaut payload specialists Congressman William Nelson and Charles Walker review hold initial version of the hand-held Vapor Diffusion Apparatus (VDA) as they discuss crystal photographic documentation flight procedure.

Prior to the US Space Shuttle Challenger disaster in early 1986, a primitive version of what was called the Vapor Diffusion Apparatus, VDA,^42^ was flown on four different space shuttle missions (Figure 1). Initially the simple VDA device consisted of a series of syringes in an aluminum frame loaded with protein solutions. Each of these extruded a 20 to 40 μl droplet of mother liquor onto its tip. The drop then equilibrated against a porous material saturated with a precipitant solution that shared its sealed chamber. This took place in individual chambers organized in trays with 24 experiment chambers. The trays were simply taped to partitions of middeck lockers of the Space Shuttle during a mission. Drop extrusion was performed manually by an astronaut using an Allen key. These first experiments were carried out principally at the instigation and direction of Professors Charles Bugg and Fred (Bud) Suddath and Larry DeLucas at the University of Alabama at Birmingham.

**Figure 2:**
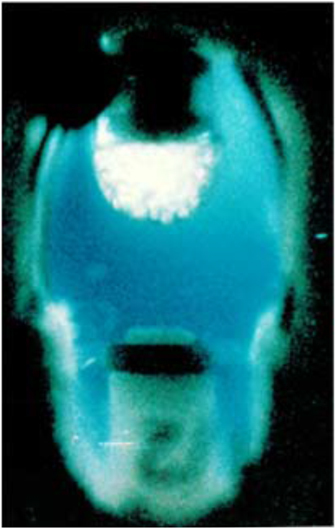
VDA syringe with protein droplet extruded on syringe tip: one of the earliest experiments on the crystallization of proteins in microgravity. (1) Used the hand-held Vapor Diffusion Apparatus (VDA). Here the protein canavalin is seen by crystallizing in a drop of mother liquor extruded into the chamber of the VDA. Porous material in the lower part of the chamber was saturated with the precipitating solution and served as the reservoir.

In spite of (or perhaps because of) the simplicity of the device, and the lack of any temperature stabilization or control, a number of different protein crystals were grown in microgravity (Figure 2). Among these were lysozyme (an enzyme), canavalin (a seed-storage protein), serum albumin (a transport protein), and a number of others whose crystallization properties were reasonably well established on the Earth. From these early experiments the conclusion emerged that crystals grown in space were of uniformly higher quality and generally of greater size.^42^ Some of the criteria on which the crystal quality conclusions were based include diffraction resolution, comparison of *I*/sigma (*I*) evaluated throughout the resolution range, and temperature factors (*B*-factors). Some preliminary X-ray diffraction measurements on canavalin and other proteins, though not definitive suggested that the crystals also diffracted to a higher resolution limit than equivalent crystals grown in the laboratory (many of these initial results were not reported due to the lack temperature control for the space experiments thus preventing precise control experiments).

**Figure 3:**
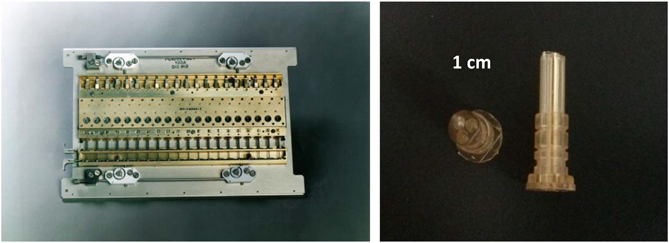
Second generation VDA. The image on the left contains 20 vapor diffusion experiment chambers (three of these trays were contained in one space shuttle incubator for a total of 60 vapor diffusion experiments. The image on the right shows a triple barrel syringe used for each experiment chamber.

Although experiments with the simple hand-held VDA came to an end with the Challenger disaster in January 1986, it served as an important precursor for the design and fabrication of a considerably more sophisticated VDA system that came into use following resumption of space shuttle missions on 26 October 1988. This enhanced VDA consisted of 60 vapor diffusion experiments maintained at constant temperature, 1 at 4 °C and 1 at 22 °C, and activated automatically by a mechanical drive that extruded protein and precipitant solution, each contained in 2 barrels of a triple-barreled syringe. The solutions were mixed by extruding them onto the syringe tip followed by withdrawing the combined solutions into a much narrower third barrel and then re-extruding them back onto the syringe tip (Figure 3). This procedure was repeated until the two solutions were thoroughly mixed. The importance of providing a mechanism for thorough mixing is critical, as partial mixing often results in regions where the precipitant concentration in contact with protein solution is exceedingly high, leading to unwanted protein precipitation. The mixed protein/precipitant solution remained on the syringe tip, thereby allowing it to equilibrate with a precipitant/reservoir solution that saturated a porous material lining the chambers. Prior to re-entry the droplet containing crystals was withdrawn into the two larger barrels and capped via an opposing plunger.

From these, and the many similar experiments which followed, numerous proteins, provided by many coinvestigators, were crystallized under a great variety of conditions by vapor diffusion. An important early paper was published in 1989 in *Science*^43^ reporting the X-ray analyses of several microgravity experiments that had produced some particularly impressive results. For the first time, it was demonstrated quantitatively that crystals of superior X-ray diffraction properties could be produced as a consequence of growth in a microgravity environment. It was shown that for several crystals at least two diffraction characteristics were improved; *I*/sigma ratio (roughly, the signal to noise ratio) over the entire resolution range, and the resolution limit itself. The significance of these results is discussed more fully below. Improved crystal size and perfection significantly increased the available X-ray data for those protein crystals, which in turn permitted a more precise definition of the molecular structures. In addition, the VDA experiments provided some of the first evidence that crystal morphology was susceptible to the influence of gravity. It should be noted that there are several uncontrollable harmful factors that interfere with microgravity protein crystal growth investigations. These include the lack of frequent access to microgravity (researchers often wait a year or more before their experiments are flown). Additional delays subsequent to the protein samples being loaded on the launch vehicle or retrieved once the samples are returned can cause unwanted protein or crystal degradation. Finally, the significant amount of documentation associated with the flight approval process combined with uncertainty regarding when an experiment will fly contribute to the lack of widespread participation by the scientific community.

**Figure 4:**
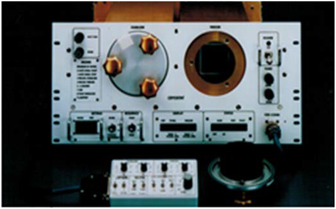
Cryostat crystallization flight hardware. This hardware was built by DARA, the German Space Agency, and provided fourteen crystal chambers maintained at controlled temperatures.

**Figure 5:**
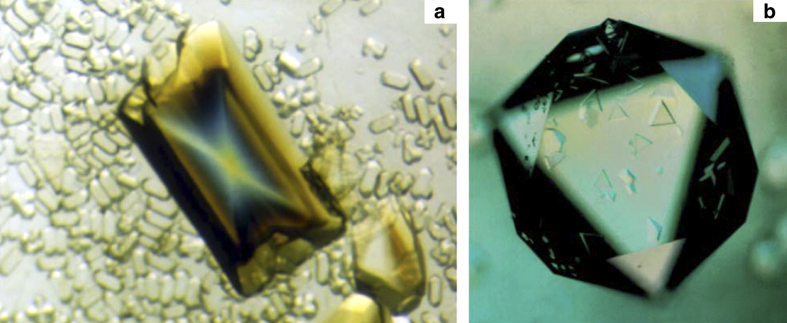
In (**a**) is an orthorhombic crystal of satellite tobacco mosaic virus (STMV) that is more than 1.5 mm in length and was about 30 times the volume of any STMV crystal ever grown on Earth. It was grown in the Cryostat instrument on IML -1. The small STMV crystals in the background formed after return to Earth when the retrograde solubility of the virus remaining in the mother liquor was induced to crystallize by the heat of the microscope lights used for observation. In (**b**) is an equivalent sized cubic crystal of the same virus, again, far exceeding in dimensions any grown in an Earth laboratory.

Concurrent with the VDA experiments, developments were continuing in Europe, and in Russia, toward more sophisticated devices using, not only vapor, but the liquid–liquid diffusion technique^44^ as well. The Cryostat device (Figure 4) was designed and built by DARA, the German Space Agency and was based on Walter Littke’s original design.^45–47^ Cryostat provided 14 crystallization chambers maintained at controlled temperatures. This system was really the first well designed and engineered apparatus for crystallization of macromolecules using direct liquid diffusion methods and, provided the first clearly successful application of that method on IML-1. In the Cryostat device, crystals of the first virus, satellite tobacco mosaic virus (STMV) like those seen in Figure 5, were obtained by liquid–liquid diffusion.

### Post-challenger results—an accumulation of data

**Figure 6:**
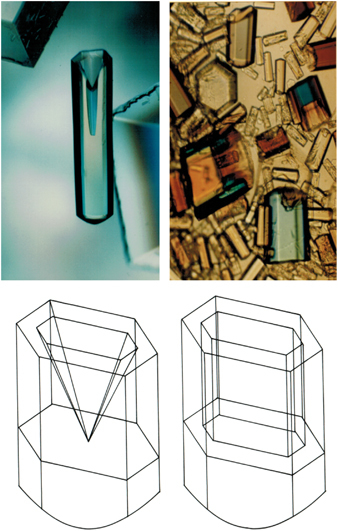
Top right is a typical hexagonal crystal of the protein canavalin grown on Earth, and equivalent crystals grown in ug on IML-2. The distinctive cusp in the Earth-grown crystal becomes a hexagonal lumen within the prismatic crystal grown in space. This is shown schematically for each below. The alteration in lumen or cusp morphology is a direct consequence of nutrient depletion in μg at the most rapidly growing face of the crystals and it illustrates the effect of the concentration gradient that persists in space. IML-2, International Microgravity Laboratory-2.

**Figure 7:**
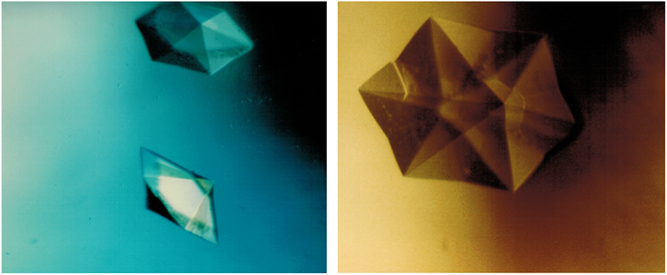
At left are hexagonal by-pyramidal crystals of turnip yellow mosaic virus (TYMV). Note the flat faces of the crystals. At right are crystals of the same virus grown in microgravity. Because of diffusion limited transport of nutrient under μg conditions, the faces are strikingly creased and the edges indented. Crystals were grown in the Advanced Protein Crystallization Facility (APCF) on IML-2. IML-2, International Microgravity Laboratory-2.

From 1988 to the present, many experiments have been conducted that produced both interesting and encouraging results. In the VDA experiments that commenced again in 1988, crystals of γ-interferon, porcine elastase, and isocitrate lyase were shown to grow larger, display more uniform morphologies, and yield diffraction data of higher resolution than equivalent crystals grown on Earth.^43^ Similar results were obtained for canavalin,^48^ and positive results continued to accumulate from this apparatus.^49^ On USML-1, experiments in the glove box using a modified vapor diffusion technique, yielded crystals of malic enzyme of substantially enhanced properties.^50,51^ On IML-1 in the Cryostat,^48,52,53^ the VDA, and on IML-2 in the European Space Agency (ESA) Advanced Protein Crystallization Facility, APCF (which also supported liquid–liquid diffusion experiments) morphological alterations to crystals of canavalin, seen in Figure 6, and another larger virus, turnip yellow mosaic virus (TYMV), seen in Figure 7, were clearly demonstrated.^54,55^

**Figure 8:**
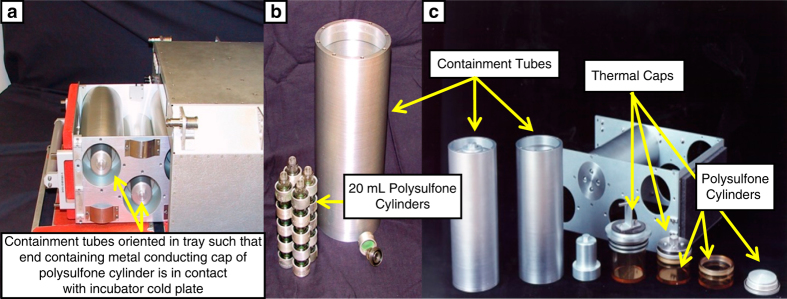
(**a**–**c**) Protein Crystallization Facility (PCF): (**a**) hardware inserted into containment tray that slides into incubator containing a cold plate that contacts the metal end of crystallization cylinders; (**b**) 50-ml cylinders stacked end-to-end thereby creating different thermal gradients for each experiment; (**c**) metal containment cylinders are shown along with 500 ml, 200 ml, and 100 ml crystallization cylinders and metal caps.

A third technique, temperature-induced batch crystallization (Figure 8), was reported to produce larger, higher resolution crystals of insulin and interferon.^56^ The complete lack of temperature-induced convection in a microgravity environment provides a clear advantage for crystallization of proteins amenable to thermally dependent crystallization. Other US investigators carried out protein crystal growth experiments on the Russian Space Station Mir, using vapor diffusion-based crystallization devices,^57–59^ including ribosomes,^60^ as have Europeans.^61–63^ In agreement with other results cited above, the Americans reported that experiments on 5 of 21 proteins produced results superior to those obtained on Earth.^58,59^

An intriguing aspect of the results accumulated from multiple missions was that when the same protein was crystallized by a variety of different techniques in microgravity, a range of crystalline products could be produced,^52,64^ pointing to the need for serial flight experiments with incremental optimization. The experiments, in sum, provided persuasive evidence that growth in microgravity could produce protein crystals of larger size, better morphology, and higher quality than were obtained on Earth. In addition, they also showed that benefits from microgravity crystal growth could, in some cases, be crucial to success in protein structure determination.^65^

Some experiments carried out by a group of European investigators using the INTOSPACE facilities and the Russian Cosima space carrier were indeterminate according to their report^61,63^ although several interesting observations emerged. A number of different proteins were crystallized, in some cases with positive benefit, whereas for others the contrary was true.^62^ Similarly ambiguous results were obtained as well from experiments carried out on the Russian Space Station. Some proteins apparently crystallized with improved characteristics and others showed no effects.^58^ A difficulty with some of these studies, however, was that there was generally no detailed quantitative analysis of the crystals carried out with regard to size distribution, morphology, defect density, or X-ray diffraction characteristics.

**Figure 9:**
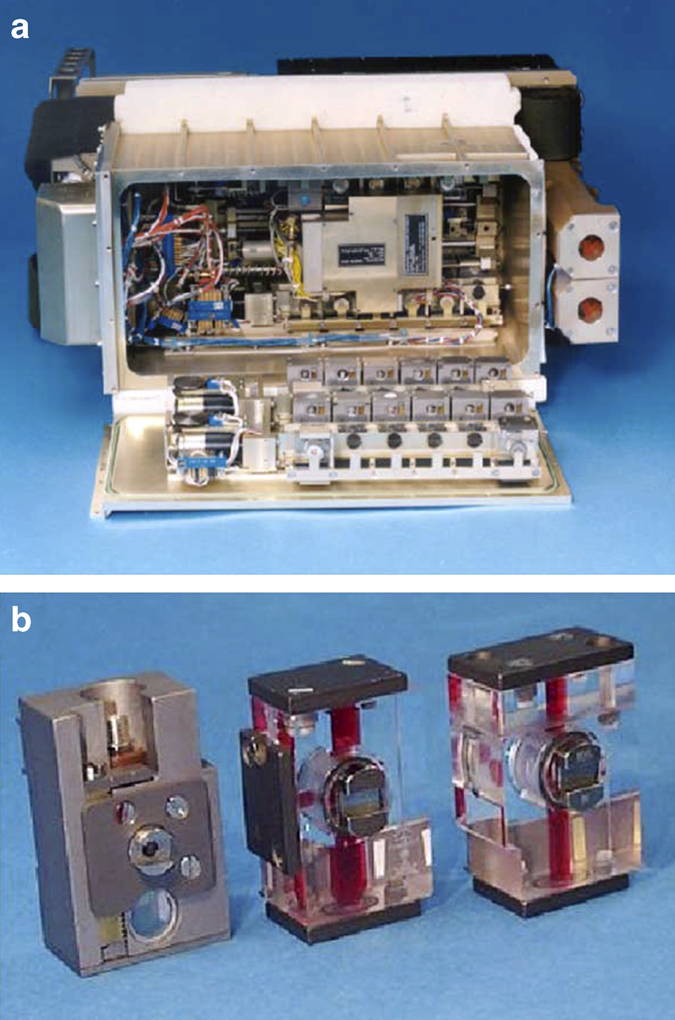
Advanced Protein Crystallization Facility; (**a**) shows the entire facility which accommodates 48 experimental reactors and (**b**) shows individual reactors for vapor, liquid and dialysis crystallization techniques.

International Microgravity Laboratory-2 (IML-2) afforded the unique opportunity to compare, on a single space mission, crystallization of identical samples in two different types of apparatus, one based on vapor diffusion apparatus (VDA) and the other (European APCF) on liquid–liquid diffusion.^54,55^ The growth of crystals of canavalin, which had been investigated a number of times previously, and the first virus crystallization attempted in space, satellite tobacco mosaic virus (STMV), were both crystallized during the 13-day mission in both hardware systems. The results from IML-1 were presented in detail^52,64^ and they provided some of the more compelling evidence that, not only did gravity influence the macromolecular crystallization process, but that the methodology employed also played a major role. The results obtained from both the VDA and the European Advanced Protein Crystallization Facility (Figure 9) were clearly positive, but they were also of a different nature.^55^

There are currently four broad criteria that are considered in comparing crystals grown in microgravity with equivalent crystals grown in Earth laboratories. These are (1) subjective visual quality, (2) maximum size and size distribution, (3) morphology, and (4) X-ray diffraction qualities. The crystals grown on IML-1 were clearly different than ground-based controls in all categories. In the case of STMV grown in both the VDA and the CRYOSTAT, the crystals were uniformly more perfect as judged by visual inspection, but in the case of the CRYOSTAT grown crystals they were more than 15 times the volume of the Earth-grown crystals. Furthermore, X-ray analysis demonstrated that the crystals from CRYOSTAT diffracted to beyond 1.8-Å resolution using conventional X-ray sources, compared with laboratory crystals that diffracted to no more than a resolution of 2.3 Å. In addition, for 15 different microgravity grown crystals, the *I*/sigma ratio was significantly better than for those from the best ground grown control crystals, and this improvement extended over the entire resolution range.

As a consequence of the extended resolution and improved signal to noise for the data, an additional 50% more X-ray intensities were available. This ultimately permitted refinement of the structure to a higher resolution than has been obtained for any other virus.^66^ This provided the most striking example to date of how the growth of macromolecular crystals in microgravity can impact the quality and precision of X-ray diffraction analyses.

Canavalin crystals grown in both the VDA and the APCF^55^ were uniformly very small, but those grown in microgravity by liquid–liquid diffusion (Figure 6) displayed a remarkable variation in their morphology when compared with their ground-grown counterparts. As shown diagrammatically in Figure 6, these hexagonal canavalin crystals, of prismatic habit, characteristically have conical occlusions along their central axes when growing, and this normally fills in by the time the crystals are mature. The crystals grown in space, however, had a conical cusp along their centers, but a finely etched hexagonal lumen whose sides appeared almost parallel with the external faces. This was not an observation limited to a few of the crystals grown in microgravity, but was characteristic of all.

A second compelling example from the same experiment revealed a unique scalloping of edges and indentation of faces on hexagonal crystals of TYMV, the largest macromolecule ever crystallized in space (280-Å diameter, 3.5 million molecular weight). The scalloping effect was clearly a consequence of the alteration of transport in microgravity (Figure 7).^54,55^

**Figure 10:**
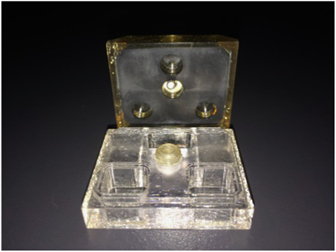
Glove box crystallization hardware.

An unusual, and ultimately very profitable, opportunity for macromolecular crystal growth in microgravity appeared with the Space Shuttle mission United States Microgravity Laboratory-1. The payload specialist on this flight of nearly 2-week duration was Dr Lawrence DeLucas, a protein crystal growth expert and X-ray crystallographer. On this mission, he was able to perform an extended series of protein crystallization experiments, numbering in the hundreds, in an enclosed glove box. The great advantage to this investigation was that Dr DeLucas could conduct crystallization trials, evaluate results, and repeat the experiments in space with altered, and incrementally improved, conditions. The availability of the glove box enabled astronauts to microscopically observe the growing crystals and then using stock solutions and syringes, adjust solution parameters (i.e., precipitant and protein concentration) in an attempt to optimize crystallization conditions. Once the solutions were prepared the top portion (contained three wells for protein solution) is combined with the bottom portion (contained three reservoir solutions) thereby sealing the protein chambers over their respective reservoir chambers and therefore initiating the vapor diffusion process (Figure 10). From this mission a number of positive results were obtained for a variety of macromolecules.^50,51^

At the same time that vapor diffusion investigations were being carried out, experiments in collaboration with scientists at Schering-Plough Pharmaceutical Company on the crystallization of interferon α-2b by application of thermal gradients were conducted on a number of missions. In these experiments, large volumes of mother liquor ranging from 50 to 500 ml were employed using flight hardware designed to produce a temperature gradient down the long axis of nonconducting cylindrical polysulfone containers (Figures 8a–c). This was accomplished by incorporating a metal thermal-conducting cap on one end of the polysulfone cylinder with the cap affixed to a cold plate within an incubator. As the temperature of the plate was automatically raised or lowered (depending on the solubility characteristics of the protein) a thermal gradient was created down the long axis of the cylinder. Crystals were consistently obtained of uniformly greater size and improved habit, as shown by morphometric analysis.^56^ This provided support for the idea that bulk crystallization of commercially valuable proteins in space might indeed have useful application. A second crystallization experiment for interferon α-2b was derived from a protein therapeutic for treatment of hepatitis C (as opposed in producing crystals for X-ray diffraction and structure determination). A solution formulation of the protein rapidly clears upon injection resulting in an effective therapeutic half-life of only a few hours. Schering-Plough was investigating use of crystalline interferon α-2b for hepatitis C treatment as a subcutaneous depot formulation, where interferon α-2b crystals slowly dissolve at the injection site and have significantly longer half-life (thus acting as a time-release drug). Unfortunately, laboratory-grown crystals tend to exhibit a broad size distribution thereby precluding their use as an injectable therapeutic. Thus, in this case the goal was to adjust the chemical conditions and the thermal gradient to grow small interferon α-2b crystals in microgravity that would hopefully exhibit a more uniform size distribution (with sizes between 1.0 and 10.0 microns). The microgravity experiments resulted in a uniform population of micron-sized crystals that met the injectable criteria. These crystals, used in primate studies at Schering-Plough, demonstrated a significant improvement in the half-life for effective drug serum levels (unpublished data).

It should be noted that not all microgravity experiments have required long durations such as those provided by the US Space Shuttle or Space Stations. For example, L. Sjolin and his colleagues in Sweden have reported the rapid, seeded, growth of crystals of Ribonuclease S aboard a sounding rocket with a mission that afforded at most 30 min of microgravity. From these crystals they nonetheless, reported extension of the diffraction analysis to a significantly higher resolution than had been previously obtained.^67^ There are, however, few proteins that can be reliably crystallized in such a short time period. Thus, this approach to microgravity has not seen much interest.

In the interval between the flight of IML-1 and its sequel IML-2, the European Space Agency, along with Dornier Aerospace, designed and built the APCF that accommodated 48 crystal growth reactors (Figure 9) in a thermally controlled chamber.^68^ The design was such that individual reactors could utilize any of three crystal growth techniques, vapor diffusion, liquid–liquid diffusion, or dialysis. Of the 48 reactors, 12 could be observed periodically and recorded by video microscopy.

**Figure 11:**
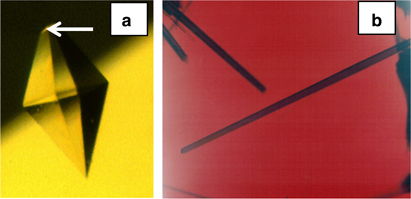
Thaumatin (**a**) and tunafish cytochrome c (**b**) crystals grown in the Advanced Protein Crystallization Facility.

Flights of APCF included two entire units totaling 96 reactors, providing opportunities for a host of European and American investigators and resulting in a bounteous yield of protein crystals. In addition, the samples provided fascinating observations^55^ regarding the resiliency of macromolecular crystals in their mother liquors in response to the stresses of flight and reentry (Figure 11). Figure 11a is a tetragonal crystal of the “sweet” protein thaumatin grown by liquid–liquid diffusion in the ESA sponsored but NASA associated APCF on International Microgravity Laboratory-2 (IML-2) by McPherson and colleagues at the University of California, Irvine. The crystal is ~1 mm in length. The truncated end of the crystal, denoted by an arrow in Figure 11a, is the point at which the crystal nucleated on the glass wall of the crystallization cell, where it remained until recovered in an Earth laboratory following the space shuttle landing. Figure 11b shows a long needle-like crystal of the protein cytochrome c from tuna fish, also grown on IML-2 in the APCF. The crystal is about 5-mm long and even after experiencing the rigors of Shuttle re-entry it also remained attached to the glass wall at one end where it nucleated.

**Figure 12:**
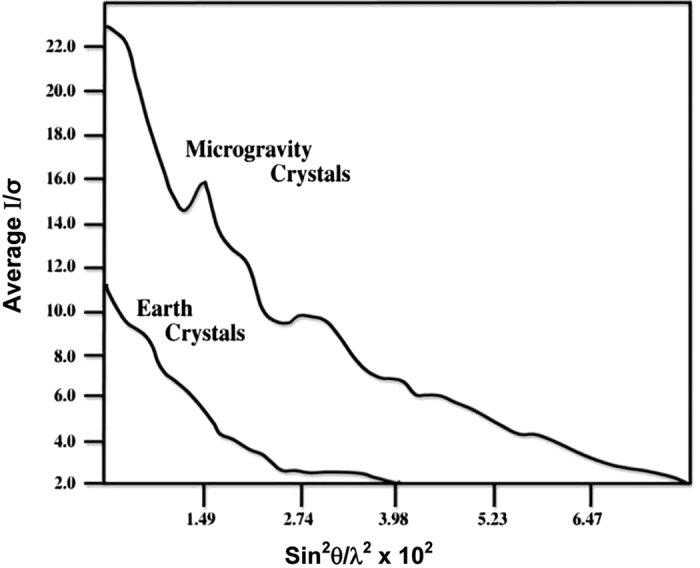
Comparison of intensity versus resolution for satellite tobacco mosaic virus. The intensity to sigma ratio for X-ray diffraction data for both Earth and microgravity-grown crystals is plotted as a function of shells of equal size and increasing resolution. The estimated s.d. (sigmas) were based on the deviations from the mean of symmetry-related and redundant measurements of individual reflections.

While positive results from various laboratories were presented at conferences but unfortunately, never found their way into the literature, some impressive findings were documented.^55^ In the APCF, for example, cubic crystals of STMV were grown by liquid–liquid diffusion in several reactors (Figure 5). These crystals exhibited >30 times the volume of equivalent crystals grown on Earth. The cubic crystal form of STMV, when grown on Earth, was a poorly diffracting crystal with a resolution limit of about 6 Å. The corresponding limit for the microgravity grown crystals was 4 Å, with an improvement in the *I*/sigma ratio over the entire resolution range (Figure 12). Although not of larger size than crystals previously grown in the laboratory, crystals grown by liquid–liquid diffusion in the APCF of both the rhombohedral and the hexagonal form of the protein canavalin demonstrated a substantially higher resolution limit. As illustrated by comparative Wilson plots,^69,70^ these increased from 2.8 to 2.3 Å, and 2.7 to 2.2 Å, respectively. Because of a significantly improved *I*/sigma ratio for all reflections, these crystals provided nearly twice the measurable X-ray diffraction data compared with crystals previously grown in the laboratory, or in other space experiments as well.

**Figure 13:**
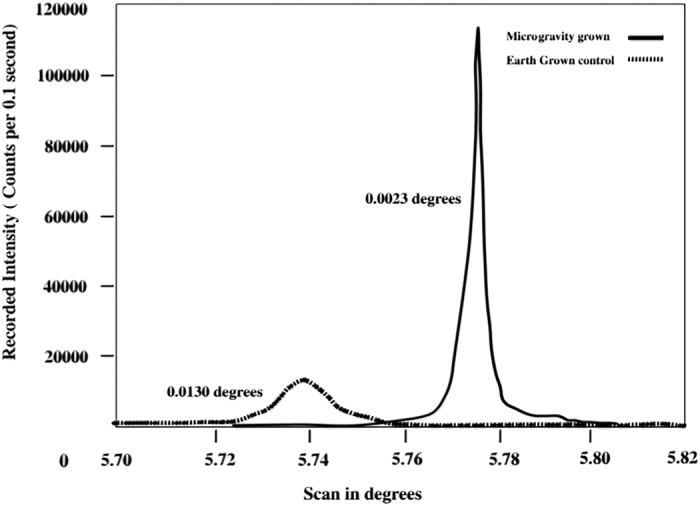
A comparison of the rocking curves for crystals of hen egg white lysozyme grown both on Earth and in microgravity in the Advanced Protein Crystallization Facility (APCF) aboard the US Space Shuttle. The much sharper peak on the right compared with the diffuse peak on the left clearly shows the microgravity lysozyme crystals to be superior in terms of mosaic spread.

In addition to the quantitative X-ray diffraction studies described above, another important finding using X-rays was reported by J. Helliwell of Oxford University. This was based on an analysis of the half width of rocking curves of intensities produced by lysozyme crystals grown, also by liquid–liquid diffusion, on IML-2 (Figure 13). He found that the rocking curves were significantly narrower than those from equivalent crystals grown on the ground.^71^ These measurements had profound implications regarding the degree of order and perfection of the crystals. They provided additional, quantitative evidence that macromolecular crystals grown in space exhibited greater inherent order and perfection. A parallel study arriving at the same conclusions was carried out later on thaumatin crystals grown in microgravity.^72^

The US Space Shuttle does not reliably provide a quiescent environment for protein crystallization. There are perturbations in the microgravity level due to crew activity, and Vernier rocket activity (used to maintain the shuttle’s speed and altitude as it circles the Earth). Activities such as these cause unwanted g-jitter and accelerations, both of which cause growing crystals to move within their growth solutions. Dr Juan Garcia-Ruiz *et al.* studied the relationship between microgravity perturbations versus crystal movement and their effect on crystal growth rate and quality.^73,74^

Although most of the experiments described to this point were carried out on the US Space Shuttle and restricted the time period over which crystals could be grown, some long-term crystallization experiments were later carried out on space stations using somewhat different, and interesting approaches. An extensive series of microgravity crystallization investigations were performed by American investigators first on the Russian Space Station Mir, and then continued on the ISS. Some results from these experiments and a more complete description of the approach and device were previously published.^75^ The idea for the experiments grew out of NASA’s need for an experiment that required no electrical or other Mir resources, required no astronaut attention, and required no significant financial resources to create. That is, it had to utilize NASA off the shelf hardware. The need was met by the simple expedient of making a biphasic “popsicle” of a protein solution directly, physically apposed to a precipitant solution in a short plastic tube and cryogenically freezing them. Literally hundreds of such samples could be made well in advance of a space mission and kept frozen and stable until flight. The samples were ultimately loaded into a NASA supplied liquid nitrogen Dewar, which maintained the samples frozen until transfer to the space station. At that point the frozen, biphasic samples thawed, thereby causing the two liquids to diffuse into one another in microgravity, and crystallization commenced.

**Figure 14:**
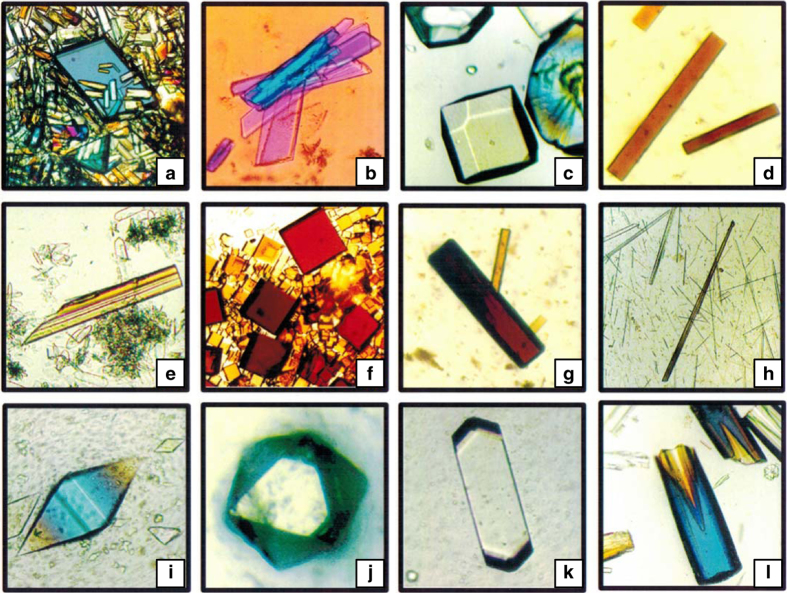
An array of crystals grown in the dewar device that used liquid–liquid diffusion from frozen biphasic samples. This experiment was performed by American investigators (Koszelak *et al.*^75^) on the Russian Space Station Mir. The crystals (labeled by row from left to right) are of top row: (**a**) rhombohedral canavalin, (**b**) creatine kinase, (**c**) lysozyme, (**d**) beef catalase; middle row: (**e**) porcine alpha amylase, (**f**) fungal catalase, (**g**) myglobin, (**h**) concanavalin B; and bottom row: (**i**) thaumatin, (**j**) apoferritin, (**k**) satellite tobacco mosaic virus (STMV), (**l**) hexagonal canavalin.

The experiments were quite successful and a vast number of crystals of different proteins, some of which are shown in Figure 14, were obtained. The Mir and later ISS experiments using this system were particularly valuable in another sense, as they were coupled with a NASA educational outreach program. High school students made the biphasic, cryofrozen flight samples in instructive “workshops” where they learned the science behind the experiments but were also able to directly participate in the space research. The educational benefits were substantial and the results from space at the same time provided many samples that became available for X-ray analysis on Earth.

**Figure 15:**
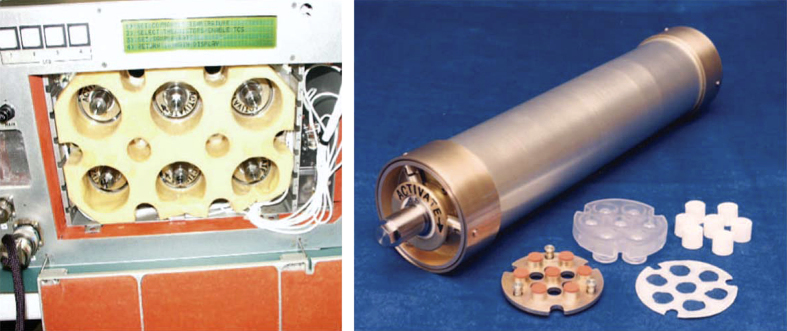
Protein Crystallization Apparatus for Microgravity (PCAM) in the typical flight configuration in an incubator containing a total of 378 experiments. Crystal growth interfaces were activated or deactivated in a flight cylinder arrangement. Each cylinder contained 9 trays with 7 experiments per tray for a total of 63 experiments per cylinder. PCAM also flew as an ambient stowage item on several flights. Individual cylinders are activated and deactivated by the crew.

Dr Daniel Carter designed two different liquid-diffusion hardware systems referred to as Protein Crystallization Apparatus for Microgravity (PCAM) and DCAM, both of which resulted in improved crystals for a variety of different investigator’s proteins.^76^ The PCAM is a multiuser vapor diffusion based crystal growth system (Figure 15). PCAM was the first example of a disposable interface flight hardware system. It featured a disposable cassette hardware interface that allowed the crystals to be photographed *in situ* and directly distributed to the coinvestigators without extensive documentation and disturbing the crystal samples. The design also allowed for rapid preflight loading and reloading and post-flight distribution. This hardware flew successfully on numerous Shuttle and ISS missions.

**Figure 16:**
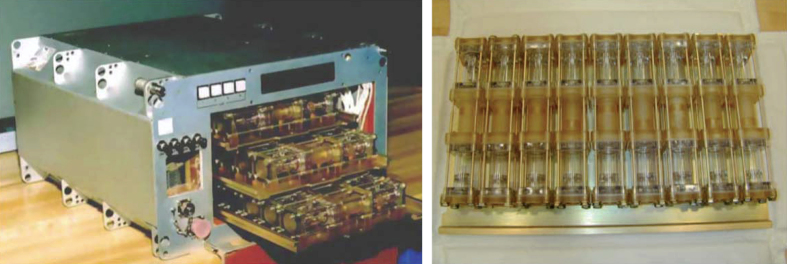
Liquid**-**Diffusion-Controlled Crystallization Apparatus for Microgravity (DCAM) is a multiuser diffusion-controlled crystallization apparatus which provided passive pre-programmed individual control of rate of approach to supersaturation in both bulk and dialysis experiments. Since each experiment was individually programmed and passively controlled, no crew interaction is required.

The DCAM, (Diffusion-controlled Crystallization Apparatus for Microgravity) proved to be especially useful for the production of extremely large centimeter size crystals for neutron diffraction and was uniquely suited for long-duration flight opportunities (Figure 16). As for the PCAM, this hardware featured a disposable flight hardware interface. This hardware flew successfully on a series of Mir, Space Shuttle, and ISS missions. In 2002, coinvestigator Dr Gerry Bunick and colleagues grew crystals of glucose isomerase in the DCAM that were used to commission the Los Alamos neutron beamline for biological diffraction (featured on the cover of the ACA Newsletter Winter, 2002 vol. 4).

**Figure 17:**
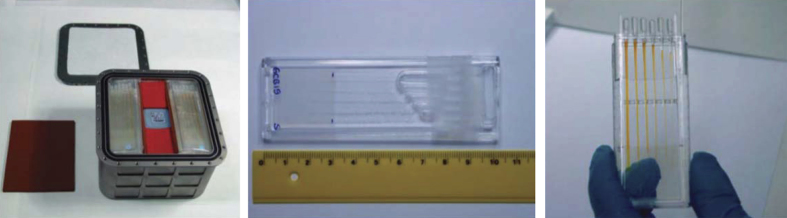
Granada Crystallization Facility. Left image shows the containment box with 138 capillaries (maximum capacity) is shown on left with middle and right images showing the capillaries in their protective containment tubes.

In 2002, another capillary crystallization system, the Granada Crystallization Facility (GCF) was developed by Dr Juan Garcia-Ruiz *et al.*^77,78^ On the basis of the concept of counter diffusion,^79^ the first and second generations of this flight hardware system were flown on multiple space missions of varying durations. The GCF external containment box measures 12.5×12.5×8.5 cm. It is capable of carrying a maximum number of 138 crystallization experiments. The capillary crystallization tubes are inserted into a protective containment sleeve as shown in Figure 17.

It is important to note that crystals grown in space represent not only common proteins useful in crystallization studies, such as canavalin and lysozyme, but other very unique macromolecules as well. These include viruses, DNA, numerous pharmaceutical targets, and a variety of membrane proteins. As additional experiments are carried out in the future, one of the most important objectives will be to determine which classes of macromolecules, and which specific members of those classes will obtain the greatest benefit from crystallization in microgravity.

### More recent research of a quantitative nature

**Figure 18:**
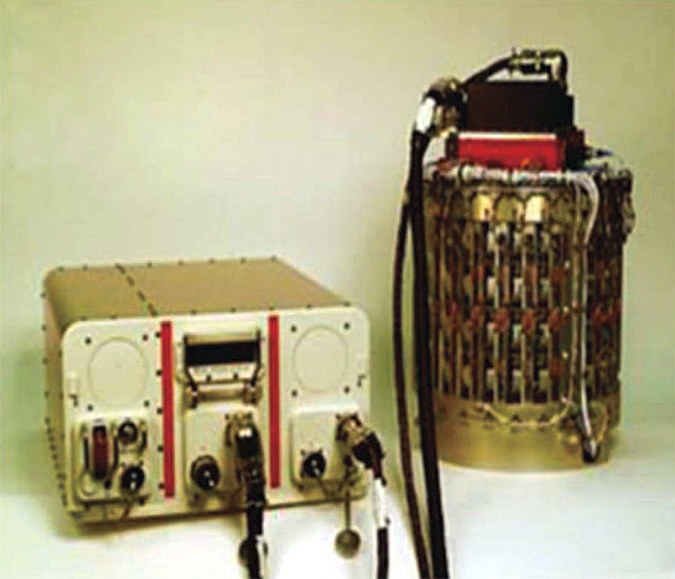
Observable Protein Crystal Growth Apparatus (OPCGA).

**Figure 19:**
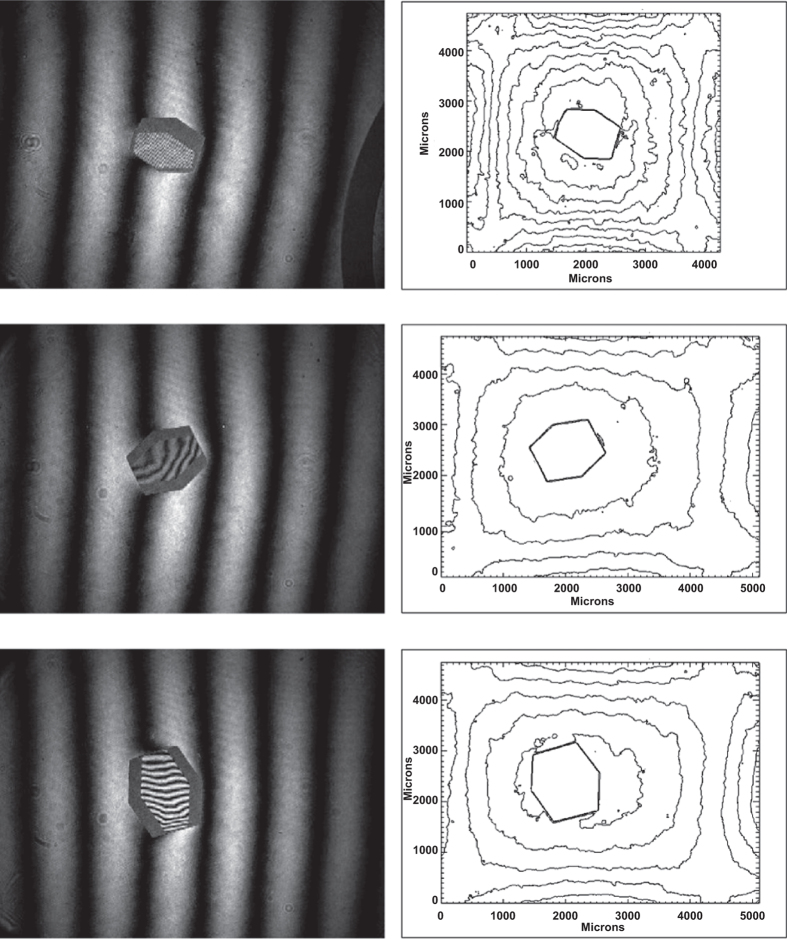
Lysozyme crystals growing in a liquid–liquid diffusion cell of the OPCGA on Earth as observed by Mach–Zehnder interferometry. Even in a 1-*g* environment, a depletion zone can be seen forming about the crystal, though it is ultimately unstable owing to convective transport in the cell. On the right each of the interferograms were quantitatively evaluated to obtain a precise description of the concentration gradient surrounding the crystal.

Virtually all experiments supported by NASA involving American scientists were focused on the comparison of microgravity-grown crystals with those obtained in laboratories on Earth, the objective being to evaluate whether the former were superior in size and quality to the latter. Little attention was given to acquiring physical data^80^ on crystals growing in microgravity that might shed light on why differences might exist. The only system specifically designed for that purpose, the Observable Protein Crystal Growth Apparatus (OPCGA)^81^ though fully constructed and tested, failed to fly as a consequence of the Columbia Space Shuttle disaster. That system contained two sophisticated, solid optics Mach–Zehnder interferometers^82^ as well as polarized light video microscopy (Figure 18). Following that misfortune, most microgravity science in low Earth orbit was abandoned for a significant time. Figure 19 shows lysozyme crystals growing in a liquid–liquid diffusion cell of the OPCGA on Earth as observed by Mach–Zehnder interferometry.

**Figure 20:**
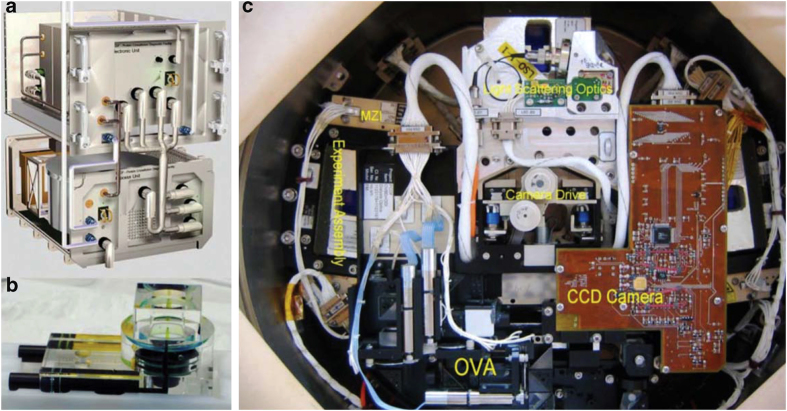
Protein Crystallization Diagnostics Facility (PCDF): (**a**) external image of flight unit, (**b**) crystallization growth cell with optical windows to support a variety of optical diagnostics, (**c**) internal view of PCDF.

At least two foreign investigations with distinctive scientific data gathering objectives continued and these yielded very encouraging and promising results that went some distance in establishing the concept of depletion zones in microgravity as responsible for observed improvement in space grown crystals. The first of these investigations, under the direction of Professor J.M. Garcia-Ruiz from Granada, Spain was sponsored by ESA using an instrument called the Protein Crystallization Diagnostics Facility that was designed and built by Dornier Aerospace, Friedrichshafen, Germany (Figure 20) Using Mach–Zhender interferometry for observation of crystals grown by liquid–liquid diffusion and counter-diffusion methods, stable depletion zones around growing crystals were visualized and recorded.^83^ Quantitative measurements were made regarding the extent of the depleted volumes and their kinetics of formation. In addition, the data suggested mixed-diffusion-interface kinetic controlled growth close to the diffusion-controlled regime.^84^ Important observations were also recorded showing the movement of crystals growing in a cell in microgravity, and the effects of microgravity instabilities during growth.^85^ Finally, the experiments validated the use of the counter-diffusion method for growing protein crystals in space, and the interferometric observations demonstrated unequivocally the existence of a self-organized supersaturation wave traveling across the diffusion reaction system.

**Figure 21:**
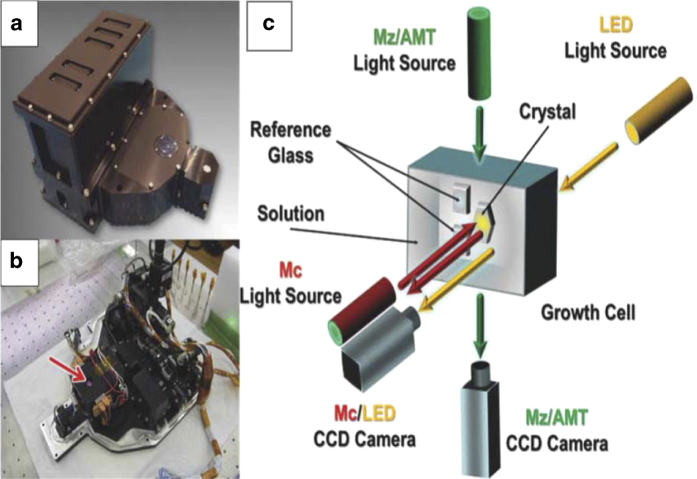
NanoStep Hardware: outer (**a**) and the inner (**b**) views of the NanoStep hardware (arrow indicates the position of the protein sample) and (**c**) diagram of Mach–Zehnder interferometer.

Interferometry was used as well in more recent experiments carried out by Professors Yoshizaki, Tsukamoto, Oshi and colleagues and sponsored by the Japan Aerospace Exploration Agency (JAXA). In these investigations, lysozyme seed crystals were used to initiate growth in microgravity and the subsequent process recorded in a way that again allowed the extraction of quantitative measures of the growth parameters.^53^ The flight hardware (designated Nanostep) consisted of a combination of Mach–Zehnder (Mz) and Michelson (EU) interferometers (Figure 21).

**Figure 22:**
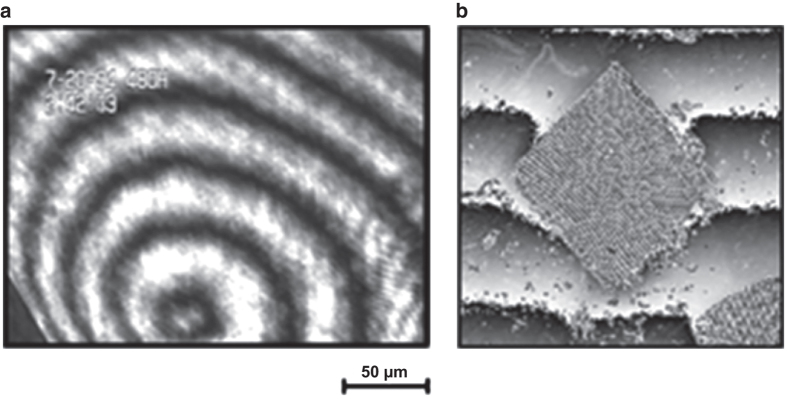
Michaelson (**a**) interferogram of the growing face of a canavalin crystal and Mach–Zehnder (**b**) interferogram showing bending of the interferometric fringes in the vicinity of a growing lysozyme crystal.

McPherson and colleagues used Michaelson Interferometry to study the solution characteristics around growing canavalin crystals. Figure 22a shows a Michaelson interferogram of the growing face of a canavalin exhibiting a single screw dislocation. Figure 22b is a Mach–Zehnder interferogram showing the bending of interferometric fringes near the active surfaces of a growing lysozyme crystal.

## Controls and comparison criteria

It can be argued that the most valid control for a crystal growth experiment in microgravity, is a parallel experiment in which portions of the identical samples flying in space are crystallized on Earth in precisely the same way, under identically the same chemical conditions, in an exactly equivalent apparatus, and during the same period that the experiment is carried out in microgravity. According to classical practices of investigation, this would seem to be the proper control. But in the case of microgravity experiments one can ask whether it is really the most appropriate, useful, accurate, and strictest control.

Most investigators believe the answer, in the case of space experiments, is no. While it may be useful, and it is probably prudent to carry out such an experiment, to evaluate carefully the effects of conditions, apparatus, and sample, this does not provide the best control. A better case can be made that because the objective is to evaluate whether, and how microgravity, *per se*, improves the inherent quality^86^ of specific macromolecular crystals, then a more stringent test is necessary to compare the crystals grown in space, and their diffraction and physical properties, with the best crystals ever grown under any conventional laboratory condition.

If one bears in mind that the comparison here is between the results of only a few, generally nonrepetitive, highly restricted, microgravity crystallization trials, against literally thousands of experiments carried out under vast arrays of conditions and methods in the laboratory, then it seems clear that there should be little in favor of the microgravity results. Even assuming that microgravity conditions and samples have been identified and chosen to be the best in the laboratory, it is still a remarkably rigorous kind of control.

A further consideration in this regard, is that a crystallization experiment carried out in space simply does not proceed in the same manner as an equivalent experiment executed in the laboratory. For example, with vapor diffusion the kinetics of the equilibration process cannot be, and are not the same in the two environments. This is suggested by theory, and has been confirmed by practice. Even more marked is the difference between a liquid–liquid diffusion experiment in a 1-*g* and microgravity environment. This was first shown in a striking manner by professor Littke’s photographs of the diffusion process as it occurred in space on a sounding rocket.^45–47,87^ An extraordinarily flat liquid front of precipitant was visualized to move evenly and without disruption into the protein solution over a period of nearly 15 min, and then, once re-entry re-established gravity, the interface disintegrated in a turbulent flurry of mixing. The former would be the experience in any space experiment, the latter in one experiment carried out in the laboratory. It is, therefore, simply not possible to use ground equivalents of space experiments as meaningful controls. At this time virtually the entire investigative community compares their space results with the best that have been recorded on the ground.

The question of what comparisons can, or should be drawn between crystals grown in microgravity and those obtained in conventional laboratories is similarly complicated. This is due in some part to our limited understanding of the physical properties of macromolecular crystals, the mechanisms by which they nucleate and grow, and a general lack of available techniques to measure their characteristics. The only quantitative method historically applied to protein, nucleic acid, and virus crystals is X-ray diffraction analysis. It now provides the principal source of data and the primary basis for quantitative comparisons of quality.

In terms of visual examination with a light microscope, generally used with polarized light, most crystals grown in the laboratory show many kinds of imperfections. Like diamonds from nature, usually only one from many is without visible defects. Most have fine cracks, striations, ill-formed faces or edges, grow in contact with other crystals, have satellites or spurs, contain inclusions, or do not extinguish polarized light well under crossed nicols. Generally, the experimentalist chooses the best and largest among them for his studies, and only 1 in a 100 grown may be actually analyzed.

One of the most striking, early observations of microgravity investigations was that an unusually large number of visually perfect crystals returned from space.^43^ Agreed, optical perfection is subjective, and as Heilbroner said, “If it’s not numbers, it’s not science; it’s opinion”. Nonetheless, a substantial number of experienced X-ray crystallographers upon seeing these first crystals and were convinced that microgravity growth of macromolecule crystals was worth further pursuit. Observations of this nature have accumulated from many investigations since, and continue to attract researcher’s interest.

Two uses have been made of the size parameter. In some experiments the largest crystals grown in space exceed, in some cases by orders of magnitude,^50–52,55,64^ the size of the largest crystals of the macromolecule ever grown in Earth’s gravity. This is clearly of significance. The important quantity is not the largest single dimension, but the total volume of the crystal. This is because the average measured intensity for all of the Bragg reflections that make up a crystal’s diffraction pattern is directly proportional to the volume of the crystal. The greater the crystal’s volume, the more unit cells there are that contribute to the Bragg reflections, and the larger the observed intensities.

A second application of the size criterion is the analysis of the size distribution of crystals grown in microgravity experiments compared with equivalent ground trials.^56^ Here the question is not whether the maximum size of the space grown crystals is greater, but whether the distribution of sizes is narrower, or whether the mean of the distribution has shifted to higher or lower values. This, as it happens, is of some importance in the pharmaceutical uses of protein crystals and protein crystallization. Space experiments using large volumes of several proteins suggest that a significant change in the size distribution can be produced in microgravity.^56^

Morphological changes, though difficult to quantitate, are often obvious. In the first well-documented paper on microgravity crystal growth, the observation was made that dendritic forms of crystals of isocitrate lyase, virtually always obtained in the laboratory, were transformed in microgravity into solid, three-dimensional forms suitable for X-ray diffraction analysis.^43^ Clear modifications in the habit, edges, faces, or general morphological character of TYMV and canavalin crystals have already been noted as a consequence of microgravity,^52,55,64^ and others have been reported from both vapor diffusion and liquid–liquid diffusion experiments carried out in a number of different instruments.^43,49–51^

Virtually all changes in morphology, with the exception of alternate polymorphs, can be ascribed to the dominance of pure diffusive transport, as it exists in microgravity, in contrast to the convective transport that dominates a 1-*g* environment. These changes provide some of the strongest, direct visual evidence that the absence of gravity does indeed affect the kinetics and the underlying development of macromolecular crystals.

X-Ray diffraction provides data for a number of kinds of analyses that allow quantitation of the relative quality of crystals grown under different conditions. The most important consideration, so far as diffraction analysis is concerned, has been assumed to be the resolution limit, or extent, of the diffraction pattern. This is usually specific and reproducible for any particular variety of macromolecular crystal, though it may vary among polymorphs, as it does for example for STMV and canavalin. It may also, of course, be dependent upon the quality and purity of the sample used to grow the crystals.

No attempt will be made here to explain the principles of X-ray crystallography and its application to macromolecules, this has been presented in detail elsewhere^88–91^ but a few points are necessary to this discussion. Every reflection in a diffraction pattern is obtained by directing a collimated beam of X-rays onto a crystal. In a simplified sense, the intensity of each diffraction peak corresponds to reflection of X-rays from a family of planes in the crystal having a characteristic interplanar spacing *d*. The d spacing is related to the X-ray scattering angle *θ* by Braggs law which is *nλ*=sin^2^*θ*/*λ* where *λ* is the wavelength of the radiation. The smaller the value of *d*, the more finely the family of planes samples the contents of the crystal, and the more detailed is the information that its diffracted intensity contains. The smaller *d*, the greater the corresponding Bragg scattering angle, theta (*θ*), and the farther from the primary X-ray beam the reflection falls in the diffraction pattern. Another way of saying this is that the farther a reflection appears from the center of the diffraction pattern, the more detailed is the information that it contributes to the structure determination.

The limit at which diffraction intensities disappear for a particular crystal defines its resolution and this, in turn determines the precision of the structural model derived from that analysis. The limit is generally far short, for macromolecular crystals, of the theoretical limit which is *λ*/2. This is owing to two causes, the inherent structural variation of the molecules that make up the crystal, and the degree of disorder of the molecules in the crystal lattice. Although there is no reasonable way that gravity could affect the conformational heterogeneity of the molecules, the order of the crystal is determined by the mechanisms and kinetics of the crystallization process, thus it is a property that we might reasonably expect to be altered by the presence or absence of gravity.

There is some disagreement as to how one measures the maximum resolution of a diffraction pattern, that is, the minimum interplanar spacing that produces diffraction. The question is when does diffraction intensity or signal, become lost in fluctuations in the background noise. Various standards have been assumed, most of these based on those traditionally used in X-ray structure refinements. In general, when the ratio of *I*/sigma declines to less than three, or in the more liberal case two, then the resolution limit is considered defined. Because microgravity experimentation is usually concerned with a comparison based on data collected from standard, or control crystals grown in the laboratory, the absolute measure is less important than consistency in how it is measured. That is, so long as the identical criterion is applied to both Earth and ground-grown crystals, the comparison should be valid.

A second quantitative criteria that has routinely been applied to comparisons of diffraction data from Earth and space crystals is the *I*/sigma ratio itself.^69,70^ If the plot of *I*/sigma is consistently higher over the entire resolution range, then this means, effectively, that the signal-to-noise ratio of the reflections that made up the diffraction pattern is uniformly enhanced, and it implies that the crystals yielding the higher ratio are of improved quality. The results shown in Figure 12 for satellite tobacco mosaic virus crystals illustrate exactly the kinds of results that are anticipated in microgravity protein crystallization experiments.

Although suggestions have been made as to why crystals grown in microgravity might produce improved *I*/sigma ratios, it is still not clear what physical property of the crystals is responsible. Although an improvement in statistical order might explain an improvement in the maximum resolution, it is not obvious that it should produce a significant change in the *I*/sigma ratio at lower resolution. It appears more likely that an improvement in the *I*/sigma ratio may reflect a reduction in the defect density of crystals grown in microgravity.^92^

Another feature of the diffraction pattern that tends to reflect the inherent order of a crystal is its mosaicity,^36,93^ and this is manifested by the width, or spread of the intensities. That is, it represents the width of the Bragg angle over which a particular family of planes in a crystal will constructively scatter X-rays. Mosaicity is in a sense a measure of the block substructure or the distribution of various kinds of physical defects in a crystal.

Early measurements of rocking curves for individual reflections of some crystals suggested that reflections produced by those grown in microgravity were narrower, thereby implying crystals having improved organization of their block structure. Recently, J. Helliwell and his colleagues at Oxford have used synchrotron radiation at Darsbury along with very sophisticated and carefully designed apparatus to measure rocking curves for crystals of lysozyme both grown in space and in the laboratory. Their results (Figure 13) clearly demonstrate a significant improvement of the latter over the former.^93^ Similar results with the same conclusions emerged from a study of microgravity-grown thaumatin crystals.^94^ Measurements such as these, though requiring skill and patience, will undoubtedly be used to an even greater extent in the future.

Other quantitative measures such as *R*-factors tend to reinforce claims that crystals grown in microgravity are of improved properties have been reported in several cases. The *R* factor is a measure of agreement between the amplitudes of the structure factors calculated from a crystallographic model and those from the original X-ray diffraction data (it is basically a measure of the goodness of fit between the model of the structure versus that obtained from the measured X-ray diffraction data) (reference). Structure refinements yielding better *R* factors and other refinement statistics^69,70,83–85,90,91,93^ and lower symmetry *R* factors for data sets have all been noted. Although these may simply be reflections of properties already discussed, they do, at the least, provide an end product evaluation of the quality of the data obtained. A difficulty, again, is that it is not straightforward to correlate these measures with physical properties or the growth mechanisms by which the crystals were obtained.

## Sources of crystal improvement in microgravity

The major question currently facing investigators is no longer whether gravity affects macromolecular crystal growth, but how does gravity affect growth? What is the mechanism by which gravity influences or intervenes in the process? Does it operate at the nucleation stage, or only to modify growth? Does it act by altering growth mechanisms, or by altering the kinetics? Does it somehow change the interaction between nutrient molecules in solution, or their rate of presentation and absorption to surfaces? Does it reduce the density of defects, change their nature, diminish the incorporation of impurities, or relieve limits to unrestricted growth? Does it do several of these simultaneously? Most current research is directed at answering these questions and identifying the means by which gravity influences macromolecular crystallization.

On the question of whether gravity affects nucleation, growth, or both, the method of Quasi Elastic Light Scattering may yield the most information because it focuses on the prenucleation stages of the process up to the appearance of microcrystals. It is conceivable that alteration of fluid properties, the occurrence of density fluctuations, or some unsuspected property such as these could affect nucleation. Indeed, any alteration in the path between starting conditions and those in the supersaturation region of the phase diagram for crystallization could have an effect on nucleation. In a liquid-diffusion experiment, buoyancy-induced convection would influence the mixing kinetics and this would most certainly affect the nucleation process.

Gravity expresses itself in fluids, including crystallization mother liquors, by altering mass transport (which in turn affects the transport of heat). Unlike nucleation, it is quite apparent that transport has a real, and in some cases profound effect on several aspects of growth. As already noted, this has been shown for conventional organic crystals, crystallization from the melt, and for macromolecular crystals themselves.^3^ This was markedly illustrated, by the direct visualization of convective plumes during the growth of lysozyme crystals by M. Pusey and his colleagues at Marshall Space Flight Center. It therefore seems reasonable to expect that it is by altering growth of crystals once a critical nucleus has formed that is important in understanding the effects of microgravity.^95^

Transport would seem to be of particular importance for macromolecular and virus crystallization because the size of the entities involved requires them to have extremely low diffusivities, two to three orders of magnitude less than for conventional molecules. Nonetheless, it is not at all clear how transport processes directly affect crystal quality, resolution of their diffraction patterns, or their final size. Indeed, previous analyses suggested that they should not.^96^ Elimination of fluid convection may, however, markedly affect the movement and distribution of macromolecules in the fluid, and their transport and absorption to crystal surfaces.

**Figure 23:**
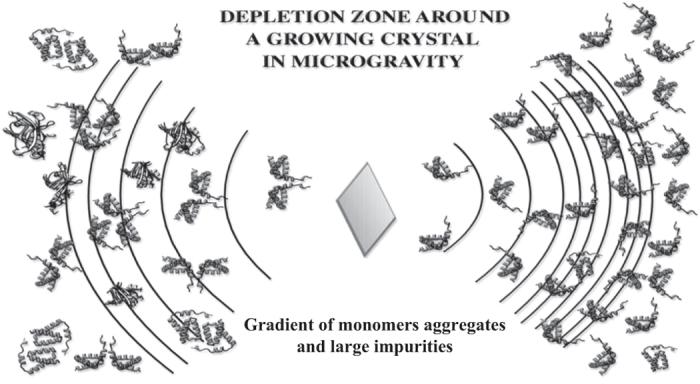
Developing protein depletion zone.

In addition, most macromolecules, particularly at high concentration, tend to form aggregates and clusters in solution of both ordered oligomeric structure, or more random, non-specific aggregates. These may very well be, for macromolecules, the major contaminants that incorporate into crystal lattices and, therefore, a major influence on the growth process. By virtue of their size, and even lower diffusivity, the movement of aggregates and large impurities in solution is even more significantly altered (Figure 23). Finally, as discussed above, some macromolecular crystals grow by the direct addition of three-dimensional nuclei and even microcrystals. All macromolecular crystals are, at least, affected by these processes. The transport of three-dimensional nuclei, again by virtue of their size, should be radically altered in the absence of gravity.

Macromolecular solutions are complex, they contain the crystallizing specie in equilibrium with higher aggregates, they contain ions, precipitants, other contaminating macromolecules, foreign particles, the walls of containing vessels, and a host of other possible impurities. By changing the transport modes in general, the specific modes for each of the individual components are altered as well. Thus, eliminating convection may superficially seem simple and straightforward, but the consequences can be broad and profound.

As discussed above, two principal effects were observed when diffraction data from microgravity grown crystals were analyzed in terms of intensity statistics as a function of Bragg angle, an extension of the resolution to higher values, and a general improvement of the data quality in all ranges of sin*θ*. In some cases, such as for STMV on IML-1, both effects have characterized the data from microgravity grown crystals^52,64^ and in others^43,48^ only the latter effect was realized.

The maximum extent of the diffraction pattern, or the resolution limit, for any protein crystal is generally thought to be a function of the inherent statistical disorder of the molecules rather than of thermal effects, which predominate in most conventional crystals. This is illustrated, for example, by the observation that lowering the temperature of a protein crystal, even to the temperature of liquid nitrogen, prolongs the crystal’s lifetime but only rarely produces an extension of its resolution limit. The statistical disorder is essentially “frozen in”.

The statistical disorder indigenous to protein crystals arises principally from two sources, the structural variability of the protein molecules that occupy the lattice points, and the distribution of the individual molecules about their lattice points. It is difficult to see, and indeed no one has argued, that microgravity should produce any improvement in the conformational homogeneity of the population of protein molecules that give rise to crystals. Thus, the improvement in the quality and resolution of X-ray data from crystals grown in microgravity must arise from enhanced order of the molecules about the crystal’s lattice points, that is, an improvement in the packing order.

We can distinguish at least two classes of disorder found in protein crystals. In the first case, all or virtually all, of the molecules in the crystal are very close to the mean orientation and lattice point position, having a scatter about the lattice points roughly that of the average statistical disorder of all of the molecules in the crystal. That is, all the molecules are reasonably ordered and the resolution limit of the X-ray data gives a measure of that average residual statistical disorder.

In the second case, there are severe defects and dislocations in protein crystals^92^ as has been clearly shown by atomic force microscopy. Molecules affected by such defects will not be slightly misaligned, but completely disordered. They will contribute virtually no constructive component to the discrete transform of the crystal, the diffraction intensities which are measured, but only to the background scatter (of which the estimated error, *σ* is a measure). Defects and dislocations, therefore, will reduce the number of usefully scattering unit cells, to which the intensity *I* is proportional, while at the same time increasing diffuse scatter, or background.

Thus, while the resolution limit of a protein crystal may be extended in microgravity by an overall improvement in the statistical order of the molecules about lattice points, as was seen for example with elastase,^43^ STMV,^52,64^ and malic enzyme^50,51^ a general improvement in the overall quality of the data as seen for canavalin^48^ and other proteins^43^ may be due to a reduction or near elimination of discrete defects and dislocations. Because defects may also be a determinant with regard to ultimate size, this may also explain the observation of generally larger crystals grown in microgravity. The question remains then as to the mechanism by which microgravity improves the general statistical order of protein crystals, i.e., their resolution limit, and how it eliminates defects and dislocations.

There are observations and data to support the contention that pronounced density driven convective flow at growing crystal surfaces introduces statistical disorder, defects, and dislocations into conventional crystals.^9,97,98^ For protein crystals, growth rates have been observed to be lower than for conventional crystals,^98–104^ but nonetheless, experience indicates that very rapidly grown protein crystals are also generally afflicted with lower order and increased defects.

Convective transport of material, as it applies to crystal growth, tends to be variable and erratic. In melt growth, for example, where convection plays a major role, it gives rise to visible growth striations. Similar effects undoubtedly occur in solution growth as well, though probably to a lesser extent. Because of the instabilities and fluctuations in transport due to convection, there will be corresponding variances in the supersaturation over regions of the crystal surface and the concentration of impurities as well. Solution composition will affect surface tension thereby altering the degree of surface-tension-driven convection (especially in vapor diffusion experiments where a solution-air interface exists). This could in turn lead to mixed growth modes, with different growth centers, or even different growth mechanisms competing with one another. This might well be expected to produce defects and discontinuities in crystals.

Lysozyme, for example, has been observed to grow by both spiral dislocations and two- dimensional nucleation simultaneously on different faces, while exhibiting normal growth on another face.^104^ It is possible that transient variations in supersaturation caused by convection could affect competition between mechanisms on an individual face, perhaps even causing cessation of one and acceleration of the other, or even determine the kind of growth mechanism that dominates growth of another face. That is, convection could produce differential effects on mechanisms as they pertain on different faces of the growing crystal. Such effects could well alter the defect density, introduce discontinuities, and modify overall morphological development. To the contrary, however, it must be pointed out that the very slow growth rate of most macromolecular crystals, compared with conventional crystals, would tend to dampen the effects of convective instabilities.

Unlike most conventional crystals, protein crystals are, in general, not initiated from seeds but are nucleated *ab initio* at very high levels of supersaturation, usually reaching 200 to 1000%. It is this high degree of supersaturation required for the nucleation of protein crystals that, in large part, distinguishes their formation and growth from that of conventional crystals. A consequence of the elevated supersaturation required for nucleation is that once a stable nucleus has formed, it must subsequently grow under very unfavorable conditions of excessive supersaturation. Distant from the metastable zone where controlled, ordered growth could occur, crystals instead accumulate molecules very rapidly, and, concomitantly, statistical disorder and a high frequency of defects. The tendency of dislocations to promote more rapid growth results in a cascade of events.

Protein crystals grow in relatively large volumes of mother liquor, hence consumption of molecules by growing crystals does not significantly exhaust the solution of protein nutrient for a long period of time. Thus, normal protein crystal growth may proceed to completion at high supersaturation and never approach the metastable phase of supersaturation where growth might proceed more favorably. In Earth’s gravity, there is continuous density driven convective mixing in the solution due to gradients arising from temperature and from incorporation of molecules by the growing crystal. The effects of diffusive transport in the laboratory are by comparison almost negligible because of the very slow rate of diffusion of large macromolecules. Because of convective mixing, protein crystals nucleated in Earth’s gravity are continuously exposed to the full concentration of protein nutrient present in the bulk solvent.

**Figure 24:**
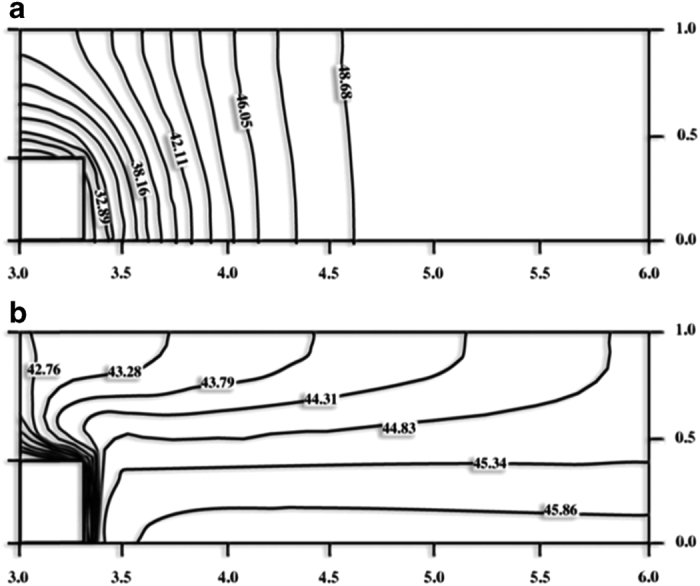
(**a**) Solution concentration gradients surrounding a growing crystal: crystallization mathematical modeling by Rosenberger and his colleagues (**b**) of the concentration gradients predicted to form around growing crystals in a microgravity environment.

Convection thus maintains, at the growing crystal interface, excessive and unfavorable supersaturation as growth proceeds. This provides an explanation as to why microgravity may significantly improve the quality of protein crystals. The mechanism for enhanced order and reduction of defects may not be directly due to convective turbulence at growing crystal surfaces, but to reduction of the concentration of nutrient in the immediate neighborhood of the growing crystals. In Figure 24 the plot at top shows that for a large, rapidly growing crystal a difference in concentration of nearly 40% may exist between the surface of the growing crystal and the bulk solvent. In the absence of gravity, there is no convective mixing of the solution and nutrient transport is dominated entirely by diffusion. It is important to emphasize, that for protein molecules, this is extremely slow.

If surfaces of some other material bound the crystallization sample, which must be, then surface-tension-driven convection (Marangoni convection) can be present as well. Convective flow from this source, however, is proportional to the change in surface tension as a function of chemical composition and temperature. Because most protein crystals are grown isothermally or at least within a narrow temperature range, temperature-induced convection is likely to be insignificant thereby limiting the microgravity surface convective forces to those affected by chemical composition.

As a macromolecule crystal forms in microgravity, a concentration gradient, or “depletion zone” is established around the nucleus. This has been suggested not only by experimental results presented above, but by mathematical models of the transport process.^105^ The concept of a depletion zone as it should exist in microgravity is shown schematically in Figure 23. As a crystal grows from solution, recruitment of molecules from the surrounding medium produces a concentration gradient, or depletion zone, that extends some distance from its surfaces. Thus the concentration of nutrient near the crystal is less than that of the bulk solution and the crystal experiences an environment of reduced supersaturation. In microgravity, because there is no convective mixing and only diffusion can act to transport molecules the depletion zones are quasi stable. The nutrient molecules (on the right) diffuse very slowly because of their large size thus prolonging and extending the effect. In addition, large impurities, which include other proteins and aggregates of the nutrient molecule (on the left) diffuse even more slowly than monomers, thus the depletion zones serves as a kind of “diffusion filter” to protect the growing crystal from incorporation of some harmful impurities. As shown in Figure 24, the developing depletion zone expands further from the crystal. Because protein diffusion is very slow, the depletion zone is quasi-stable and the net effect is that the surfaces of the growing crystal interface with a local solution phase at a significantly lower concentration of protein nutrient than exists in the bulk solvent. The crystal, as it grows, experiences a reduction in its local degree of supersaturation and essentially creates for itself an environment equivalent to the metastable region where optimal growth might be expected to occur.

It seems likely that stabilization of depletion zones and the creation of local regions of reduced supersaturation around growing protein crystals provide the primary mechanisms for an improvement of protein crystal quality by a microgravity environment. A fundamentally uncontrolled growth process in the presence of gravity becomes self-regulating in microgravity.

If this argument is indeed correct, then protein crystals demonstrating the greatest degree of improvement should be those that have the highest growth rates, and those that nucleate only at very high levels of supersaturation. We might expect similar quality enhancement in Earth’s gravity if mechanisms could be found to promote nucleation at reduced supersaturation, for example by seeding with existing crystals, or with heterogeneous and epitaxial nucleants.^106,107^ Furthermore, improvement should be expected if procedures can be devised to eliminate convective fluid transport and thereby preserve depletion zones developing around growing crystals. Crystals grown in gels or in highly viscous solutions might provide such means.^95,108–110^ A viscous gel (typically agarose gels are used) retards buoyancy-induced convective flow (particularly the movement of large macromolecules) thereby simulating the diffusion-dominated microgravity environment.

Those crystals grown in microgravity that demonstrate improved *I*/*σ* ratios for all ranges of *θ*/*λ* should contain fewer pronounced defects and dislocations. Defect density can be evaluated using electron or atomic force microscopy techniques. This would be particularly informative for crystals of large, asymmetric protein molecules. Finally, a direct examination of the depletion zones surrounding protein crystals growing in microgravity, along with controls growing in Earth’s gravity would be revealing and should, according to models,^105^ exhibit visible differences.

Although convection and convective mixing tend to dominate discussion of crystal growth in microgravity, the effects of sedimentation should not be ignored. It is clear from time lapse video recordings of protein crystallization on Earth that virtually all nuclei reaching microcrystal size immediately sediment to the bottom of their containers, whether these are glass plates, tubes, or hanging drops. As they proceed to grow, they are invariably in contact with the surfaces of their containers, and usually in contact with other growing crystals in the sample. Such contact, particularly in the latter case, may have profound effects of the morphology, degree of perfection, and ultimate size of the crystals.

This is certainly not the case in microgravity. There, crystals are seen to maintain a stable and defined position over long periods of time, to appear to arrange themselves virtually equidistant from one another, and to experience few encounters. This of course provides the most favorable solution environment for multiple growing crystals since it minimizes overlap of their respective diffusion fields and assures more or less uniform access of all faces to nutrient molecules.^111^

Atomic force microscopy (AFM) of protein and particularly virus crystal growth show even more dramatically the effect of sedimentation. For several crystals studied by AFM it was clear that three dimensional nuclei and even microcrystals continually sediment on the surfaces of growing crystals.^21,112^ These were then incorporated both with, and without the formation of visible defects. In either case, however, it is difficult to imagine that the phenomenon would be favorable to the quality of the crystals. Hence, elimination of the sedimentation of microcrystals and nuclei in a microgravity environment should provide further unique advantages to crystal growth. This should be particularly so for crystals such as those of the virus STMV, which even involve adsorbed nuclei as a principal mechanism for growth. Even for more conventional proteins such as lysozyme, however, which has also been observed to incorporate micro-nuclei, this may be of significant consequence.

By elimination of sedimentation, other technical advantages may also be obtained for growing crystals that are not available on Earth. For example, in the free interface diffusion method (see above), once a nucleus develops at the interface between two liquids, its increasing weight causes it to fall away from the interface where it was growing into a solution where it can no longer do so. Thus this technique is inherently self-limiting because of gravity. The only crystals that really do well are those that nucleate on and adhere to the walls of the containing vessel. In space, however, the liquid–liquid diffusion method works extremely well as shown now by numerous experiments.^43,45^

Another important advantage may be obtained (though to this point it has not) in the case of macromolecular crystallization from containerless processing.^113^ Using either acoustic, electrostatic or magnetic levitation and positioning methods free floating droplets of mother liquor could be deployed and maintained stationary in microgravity.^114,115^ This would completely eliminate any contact between mother liquor and crystals with a foreign surface. It might be expected that such containerless approaches would reduce heterogeneous nucleation on vessel walls and eliminate surface effects in general.

### Summary

Use of a microgravity environment to improve the quality of macromolecular crystals has been under investigation since 1985. Results from a large and diverse on a number of proteins suggest that the diffusion-dominated environment provided via different microgravity platforms is often beneficial to several different protein crystallization techniques. The research was performed by group of international investigators who were sponsored by space agencies from several countries including the National Aeronautics and Space Administration (NASA), ESA, JAXA, Canadian Space Agency (CSA), German Aerospace Center (DLR) and the China National Space Administration. In addition to the microgravity crystallization experiments these agencies also provided support for investigations that address the fundamental theoretical and experimental aspects associated with macromolecular crystallization. These investigations included the development of novel crystallization techniques and hardware that often include special diagnostic capabilities. Results from the microgravity and ground-based studies have provided a fundamental understanding of how gravity can adversely affect crystal nucleation and growth.

Although there is sufficient evidence demonstrating the potential for a microgravity environment to improve the quality of macromolecular crystals this capability has not gained widespread enthusiasm and utilization by the academic/industrial structural biology community. As noted previously, this is predominantly owing to multiple constraints associated with space research including (1) inability to utilize the microgravity environment on a regular basis, (2) launch delays that often result in sample degradation, (3) delays in retrieving crystalline samples (resulting in crystal degradation), (4) the substantial amount of paperwork associated with obtaining flight approval for protein samples, and (5) the general uncertainty related to spaceflight. The most recent NRC review of NASA’s microgravity protein crystallization program (http://www.nature.com/nature/journal/v394/n6690/full/394213b0.html) stated that results were inconclusive. The review concluded that many of the reported crystal quality improvements were incremental and noted that there were difficulties conducting appropriate control experiments. The review concluded that microgravity protein crystallization had thus far resulted in a limited effect on structural biology. It concluded by suggesting that future microgravity experiments focus on compelling biological problems where the production of diffraction-quality crystals is extremely challenging and the chief barrier preventing structural solutions. In response to this report, there are a number of microgravity investigations currently underway that involve challenging crystallizations for aqueous proteins, protein-protein complexes and membrane proteins. The growing availability of commercial vehicles capable of transporting experiments to and from the International Space Station is projected to provide bimonthly access in the not too distant future. The ability to provide frequent access should help investigators perform statistically relevant crystal quality evaluations for a variety of challenging, biologically significant proteins. Positive results from these investigations combined with more frequent access to the unique microgravity environment should attract a large group of users from academia and industry.
